# Selection and characterization of DNA aptamer for metastatic prostate cancer recognition and tissue imaging

**DOI:** 10.18632/oncotarget.9262

**Published:** 2016-05-10

**Authors:** Minlan Duan, Yuqian Long, Cai Yang, Xiaoqiu Wu, Yang Sun, Jianglin Li, Xiaoxiao Hu, Wei Lin, Dongmei Han, Yifan Zhao, Jing Liu, Mao Ye, Weihong Tan

**Affiliations:** ^1^ Molecular Science and Biomedicine Laboratory, State Key Laboratory for Chemo/Biosensing and Chemometrics, College of Biology, College of Chemistry and Chemical Engineering, Collaborative Innovation Center for Molecular Engineering for Theranostics, Hunan University, Changsha, Hunan 410082, China; ^2^ School of Life Sciences, State Key Laboratory of Medical Genetics, Central South University, Changsha, Hunan 410078, China; ^3^ Department of Chemistry, Center for Research at Bio/Nano Interface, University Health Cancer Center, University of Florida Genetics Institute and McKnight Brain Institute, University of Florida, Gainesville, FL 32611, USA; ^4^ Department of Physiology and Functional Genomics, University of Florida, Gainesville, FL 32611, USA

**Keywords:** prostate cancer, aptamer, SELEX, metastasis, binding affinity

## Abstract

Prostate cancer (PCa) is the second leading cause of death and most prevalent cancer in men. The absence of curative options for castration-resistant metastatic prostate cancer and biomarkers able to discriminate between indolent and aggressive tumors contribute to these statistics. In this study, a DNA aptamer termed DML-7 was successfully selected against human PCa cell line DU145 by using the cell-based systematic evolution of ligands by exponential enrichment (SELEX) method. The selected aptamer DML-7 was found to internalize into target cells in a temperature-dependent manner and exhibit high binding affinity for target cells with dissociation constants in the nanomolar range. Binding analysis further revealed that DML-7 only binds to DU145 and PC-3 cells with metastatic potential, but not to LNCaP or 22Rv1 cells with low or nonmetastatic potential, demonstrating that DML-7 has excellent selectivity for the recognition of the metastatic PCa cells. Clinical tissue imaging further confirmed these results. Therefore, both high binding affinity and specificity to metastatic PCa cells and tissues afford DML-7 with the potential for development into a novel tool for diagnosis and targeted drug delivery against metastatic prostate cancer.

## INTRODUCTION

Prostate cancer (PCa) is the most common noncutaneous malignancy and the second leading cause of cancer death in men in many western countries [1]. According to the National Cancer Institute, an estimated 220,800 new cases will be diagnosed in the coming year, while an estimated 27,540 men died from prostate cancer in 2015 in the United States alone. In the early stages, prostate cancer cells rely heavily on androgens for continued oncogenic growth [2]. Accordingly, localized early-stage tumors can be treated with androgen deprivation therapy (ADT). However, patients with prostate cancer may progress to an aggressive and metastatic state refractory to androgen deprivation, thus leading to high mortality [3]. Currently, metastatic castration-resistant (androgen-independent) prostate cancer remains incurable [4].

With the increase of PCa, the detection of PCa is critically important to reduce mortality rate. Prostate-specific antigen (PSA) is the best-known biomarker, and it is typically used for screening and diagnosis of PCa [5]. However, its use is controversial based on the lack of specificity [6] in that increased PSA levels have been found in benign prostatic hyperplasia [7] and prostatitis [8]. Meanwhile, numerous factors affect PSA levels, including obesity, ejaculation, aspirin and various herbal mixtures [6]. False positives have resulted in unnecessary biopsy for healthy people and overtreatment for patients [9]. These facts call for the development of more effective biomarkers for the detection of PCa.

Aptamers are short, single-stranded DNA (ssDNA) or RNA oligonucleotides able to fold into well-defined three-dimensional structures through various intramolecular interactions, followed by the formation of stable and specific complexes with different targets through complementary shape interaction [10, 11]. Similar to protein antibodies, aptamers bind to their targets with high affinity and specificity [12]. Typically, the binding affinity of aptamers is in the nanomolar to picomolar range. However, aptamers hold several attractive properties over antibodies, such as low molecular weight, high stability, easy and reproducible synthesis, lack of immunogenicity, rapid tissue penetration, and low toxicity [13].

Aptamers are generated through an *in vitro* iterative selection process known as SELEX (systematic evolution of ligands by exponential enrichment). Cell-SELEX is a variant of the SELEX procedure using whole living cells as targets for aptamer selection [14]. Based on this method, cell-specific aptamers can be generated without any prior knowledge about target cell surface molecules, thereby allowing the aptamer's binding target to retain its native conformation. Up to now, many aptamers have been generated by cell-SELEX against various cancer cell lines, including leukemia [15], lung cancer [16], colon cancer [17], hepatocellular carcinoma [18], ovarian cancer [19], and gastric cancer [20]. These aptamers, which have been applied in biomedical research for cancer cell detection, cell capture, imaging, targeted therapy and biomarker discovery, show potential for application in early cancer diagnosis and targeted therapeutics.

In this study, we adopted the cell-SELEX strategy to obtain a DNA aptamer, termed DML-7. DML-7 binds to the classical DU145 metastatic prostate cancer cell line with high affinity and can be internalized in a temperature-dependent manner. Binding analysis revealed that DML-7 only binds to DU145 and PC-3 cells with metastatic potential, but not to LNCaP or 22Rv1 with low or nonmetastastic potential, demonstrating that DML-7 holds excellent selectivity for the recognition of the metastatic PCa cells. Clinical tissue imaging further confirmed these results. Therefore, both high binding affinity and specificity to metastatic PCa cells and tissues afford DML-7 with the potential for development into a novel tool for diagnosis and targeted drug delivery against metastatic PCa.

## RESULTS AND DISCUSSION

### Selection of DNA aptamer against PCa cell line DU145

**Figure 1:**
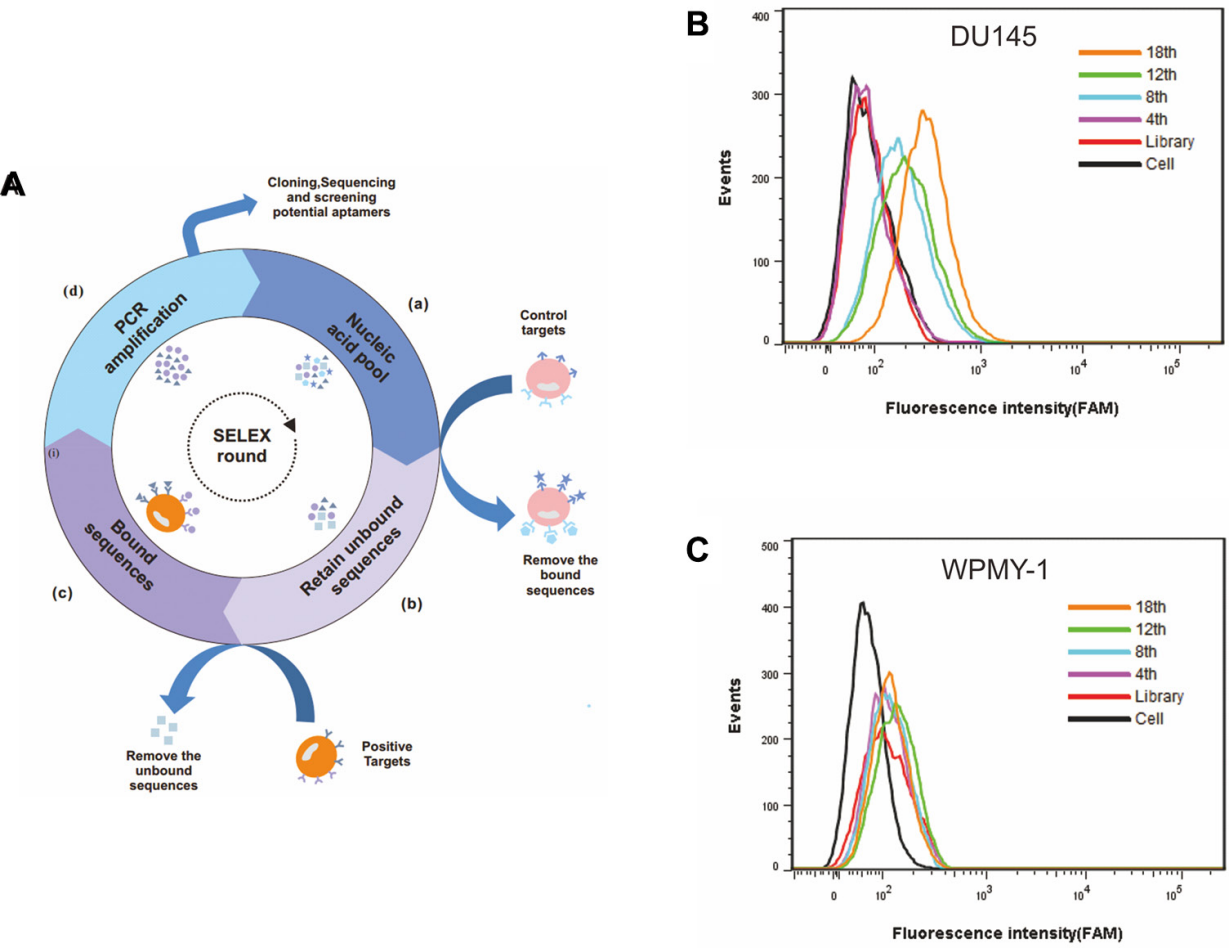
Monitoring the enrichment of cell-SELEX progression (**A**) Schematic representation of the cell-based aptamer selection. (**B**) Binding of enriched pools to DU145 cells (target cells) and (**C**) WPMY-1 cells (control cells) from the 4th, 8th, 12th and 18th rounds was monitored by flow cytometry assay. The black curve represents the background fluorescence of untreated cells. Unselected FAM-labeled DNA library was used as negative control.

To obtain aptamers against metastatic PCa cells, human DU145 cells derived from a metastatic brain cancer patient were used as target cells for positive selection. A human prostatic stromal myofibroblast cell line, WPMY-1, was used as the negative control for counter-selection to remove sequences binding to both target and control cell lines. The cell-SELEX process is illustrated in Figure 1A. For the first two selection rounds, only DU145 cells were applied for positive selection to enrich ssDNA sequences, to the extent possible, on target cells. Beginning with the third round, the ssDNA pool was first incubated with WPMY-1 cells to remove nonspecific sequences, and then unbound DNA sequences were collected and further incubated with target DU145 cells for positive selection. The ssDNA pool collected after each round of selection was amplified by PCR for next-round selection.

The enrichment of ssDNA pool during each round of selection was monitored by flow cytometry. The fluorescence intensity of the labeled cells reflected the binding capacity of enriched pools. Indeed, with increasing number of selection rounds, a steady increase in fluorescence intensity on target DU145 cells was observed (Figure 1B), indicating that ssDNA sequences with better binding affinity to DU145 cells were enriched. In contrast, no significant change of fluorescence intensity on control WPMY-1 cells was observed (Figure 1C), suggesting that the enriched ssDNA sequences were specific to DU145 cells. By the 18th round of selection, the maximum fluorescence intensity had been reached on the DU145 cells (Figure 1B).

### Identification of ssDNA aptamer candidates binding to PCa cells

**Figure 2:**
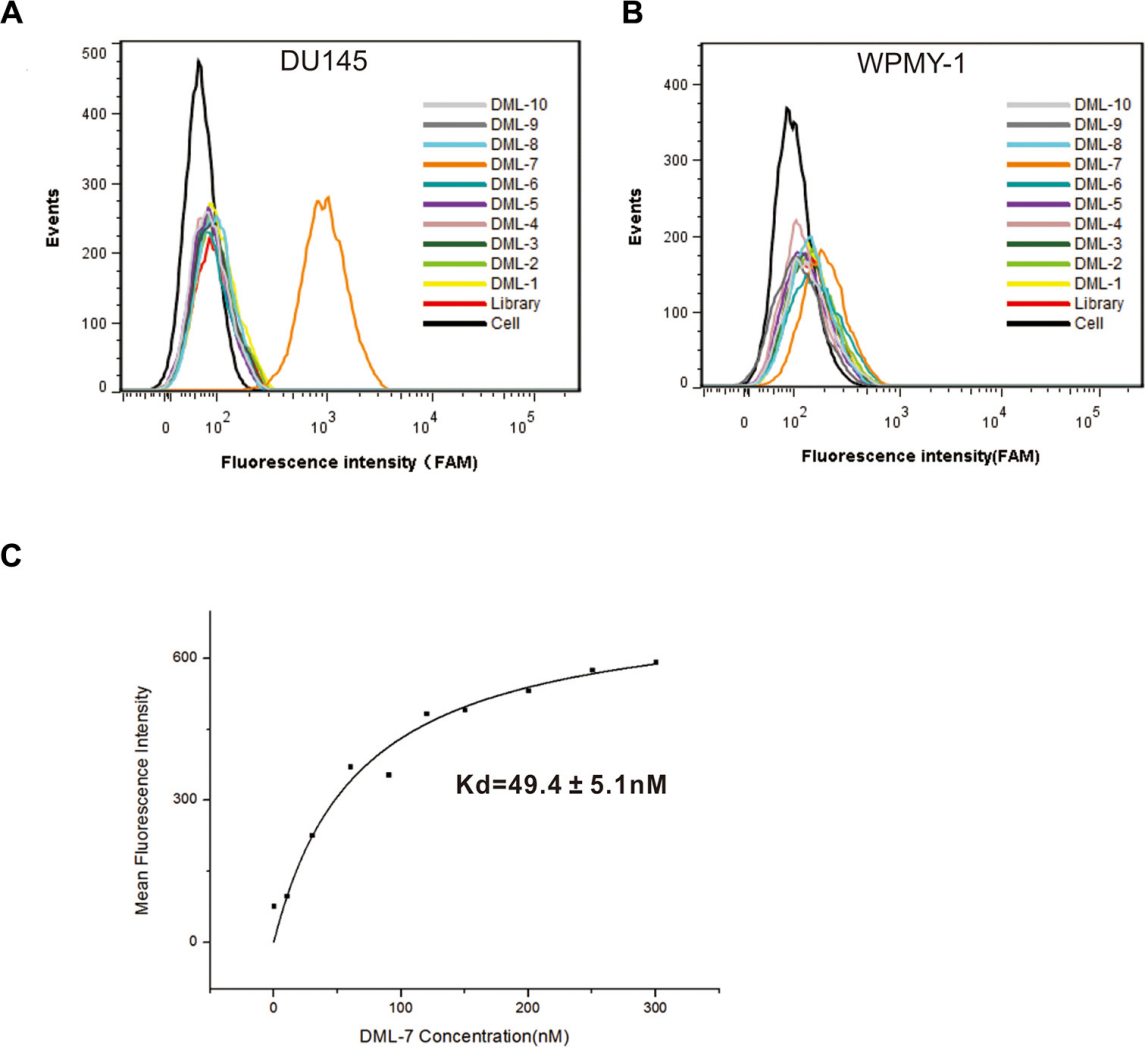
Binding assay of the selected aptamers Binding of the selected aptamers to (**A**) DU145 cells and (**B**) WPMY-1 cells was analyzed by flow cytometry. The black curve represents the background fluorescence of untreated cells. Unselected FAM-labeled DNA library was used as negative control. (**C**) Dissociation constant of DML-7 for DU145 cells was determined by flow cytometry.

To identify individual aptamer binding to DU145 cells, ssDNA pool from the 18th round was sequenced by a high-throughput sequencing device. Based on their sequential repeatability, secondary structures and homogeneity, the sequenced aptamer candidates were classified into different groups. Ten representative sequences from different groups were chosen and synthesized for further characterization (Table 1). The binding abilities of the selected sequences to target and control cells were tested with flow cytometry. Interestingly, one of ten sequences, termed DML-7, showed significant binding to DU145 cells, rather than control cells, indicating its specific recognition ability (Figure 2A and 2B). To further determine the binding affinity of DML-7 to DU145 cells, the equilibrium dissociation constant (K_*d*_) was evaluated. Accordingly, DU145 cells were incubated with different concentrations of FAM-labeled DML-7 or initial library at 4°C for 45 minutes, followed by monitoring fluorescence intensity by flow cytometry. After subtracting the geometric mean fluorescence (GMF) intensity of cells incubated with library in the highest concentration from that of cells treated with DML-7, the K_*d*_ of DML-7 for DU145 cells was calculated as Y = B max X/(K_*d*_ +X). As shown in Figure 2C, the K_*d*_ of DML-7 for DU145 cells is in the low nanomolar range, which is about 49.4 ± 5.1nM, revealing that aptamer DML-7 could bind with high affinity to its cognate target.

**Table 1:** Sequences of selected aptamers to DU145 cells

Aptamer	Sequences
DML-1	ACGCTCGGATGCCACTACAGACTCTTACTCGCCTATCTCTCTTACTCCTCCCTCTTCTGTCACCAGCACGTCCATGAG
DML-2	ACGCTCGGATGCCACTACAGAGTCTCGTCTGGTTTGCTGAGGTGGGCGACGGTGAAAAGAGTCACCAGCACGTCCATGAG
DML-3	ACGCTCGGATGCCACTACAGCAAGGTGCAAATTGAAGGGGGTGGGTTGGGATGGTTGGTGTCACCAGCACGTCCATGAG
DML-4	ACGCTCGGATGCCACTACAGTGGCATCGTGGTATCCGTCGTAGAAGAAAGTGGTGGCATGTCACCAGCACGTCCATGAG
DML-5	ACGCTCGGATGCCACTACAGGCAGGAGAGCTGATTCCGGGCGTAGAAAGTAAAATTTGTGTCACCAGCACGTCCATGAG
DML-6	ACGCTCGGATGCCACTACAGATCGGGTAATGGGCGCTCGGTAGAAATGTAGGATTGCATGGTCACCAGCACGTCCATGAG
DML-7	ACGCTCGGATGCCACTACAGGTTGGGGTCGGGCATGCGTCCGGAGAAGGGCAAACGAGAGGTCACCAGCACGTCCATGAG
DML-8	ACGCTCGGATGCCACTACAGCCCTCTCGCACTCTCTCAAATCCGAGCCATCCGATGCTTTGTCACCAGCACGTCCATGAG
DML-9	ACGCTCGGATGCCACTACAGGCAGACGAGGAGAGAGCGGTTGTATTTCGAGTGTAAAAGTGTCACCAGCACGTCCATGAG
DML-10	ACGCTCGGATGCCACTACAGTGGGTGTTCCGACATCCGAGAGCTTGAATAGTGGCGTATAGTCACCAGCACGTCCATGAG

### Determination of target type for aptamer DML-7

**Figure 3:**
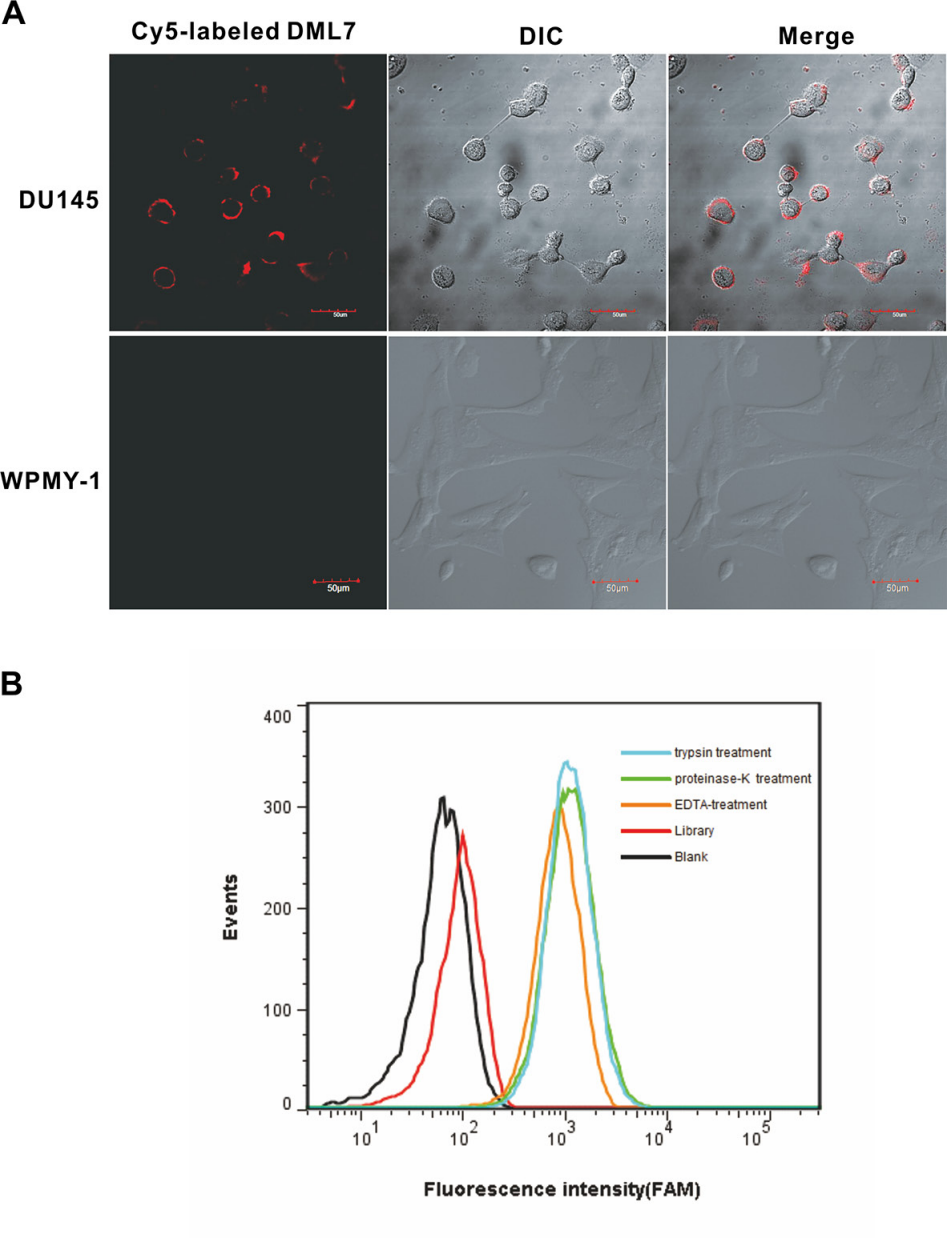
Binding site of DML-7 to DU145 cells (**A**) DU145 and WPMY-1 cells were incubated with Cy5-labeled DML-7, respectively. Fluorescence image, differential interference contrast (DIC) or merged confocal images were shown. (**B**) After treatment with trypsin, proteinase K or EDTA, the binding of DML-7 to DU145 cells was analyzed by flow cytometry.

Aptamer binding specificity and sites were further evaluated by confocal imaging using Cy5-labeled DML-7. As shown in Figure 3A, intense fluorescence was observed on the surface of DU145 cells, but not WPMY-1 cells, after incubation with DML-7. These results demonstrate that DML-7 could selectively bind to the surface of DU145 cells. However, to accurately elucidate the cell surface receptor for this aptamer ligand, DU145 cells were treated with proteinase K or trypsin for a short time respectively, before incubating the aptamer with the treated cells. Neither treatment caused DML-7 to lose its binding ability to DU145 cells (Figure 3B). The fact that proteinases, such as proteinase K and trypsin, can remove membrane proteins on the cell surface seems to suggest that aptamer DML-7 does not target membrane proteins. However, it is worth noting that some membrane proteins are highly resistant to the digestion with trypsin or proteinase K [21, 22]. Also, post-translational modification of proteins, such as glycosylation, often limits proteinase access to cleavage sites, thus reinforcing resistance to proteinase digestion [23, 24]. Therefore, identifying the receptor for this aptamer ligand remains an open question worthy of further investigation.

### Internalization of DML-7 into DU145 cells

**Figure 4:**
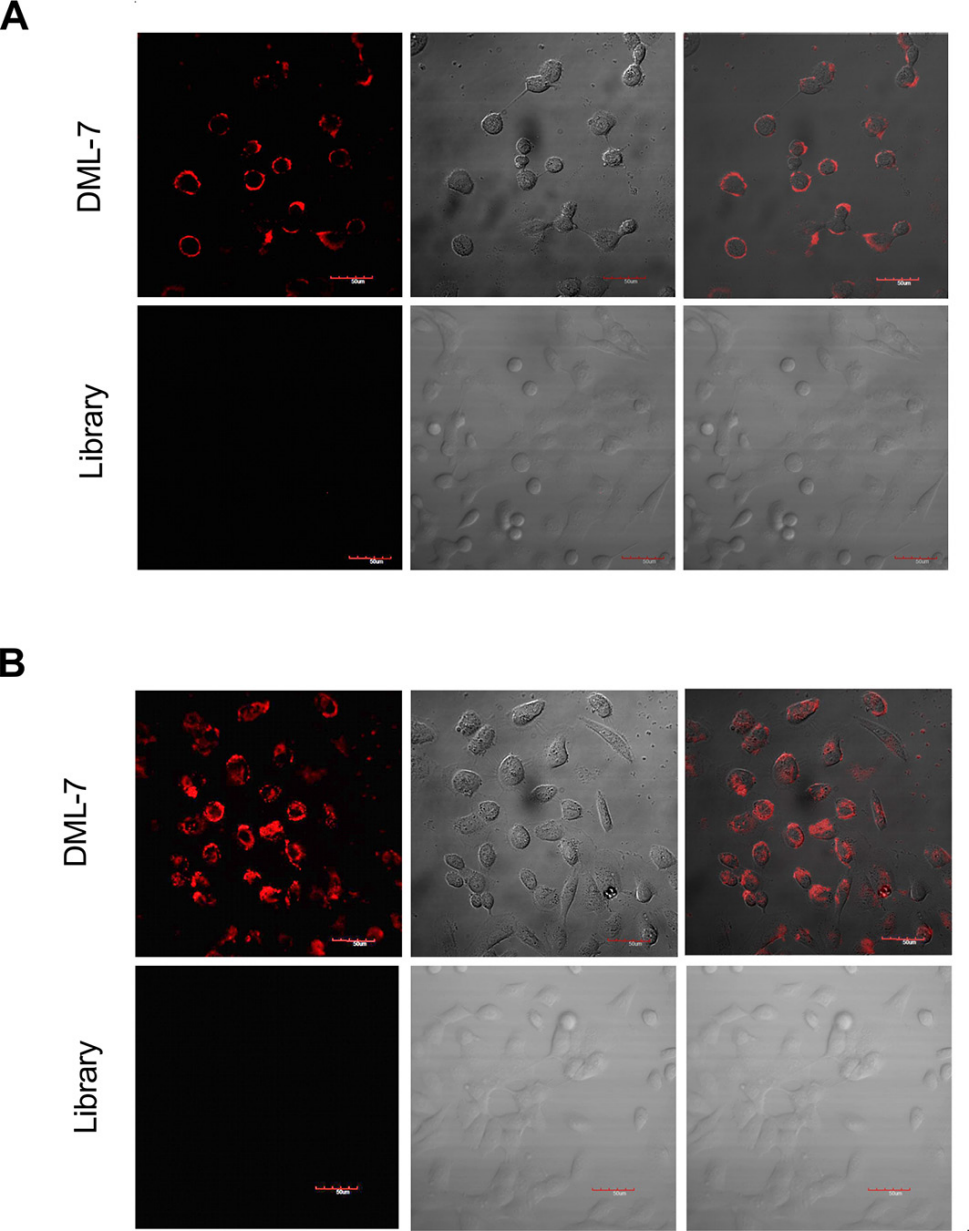
Internalization of DML-7 into DU145 cells (**A**) DU145 cells were incubated with Cy5-labeled DML-7 and library at (A) 4°C or (**B**) 37°C, respectively. Fluorescence image, differential interference contrast (DIC) or merged confocal images were shown.

Studies have shown that some aptamers can be internalized inside living cells by endocytosis [25, 26]. Internalization is very important for the application of aptamers *in vivo*, such as targeted drug delivery. Therefore, to investigate the internalization capacity of DML-7, DU145 cells were incubated with Cy5-labeled DML-7 and library at 4°C and 37°C for 2 h, respectively. Strong fluorescence was observed on the periphery of DU145 cells after incubation with DML-7 at 4°C. However, after incubation at 37°C, the fluorescence signal originated from inside the cells, indicating that DML-7 had entered cells. In contrast, DU145 cells displayed no significant fluorescence signal when incubated with the unselected DNA library at either 4°C or 37°C (Figure 4A and 4B). These results demonstrated that DML-7 can be internalized into DU145 cells in a temperature-dependent manner.

### Binding characterization of DML-7 sequence

**Figure 5:**
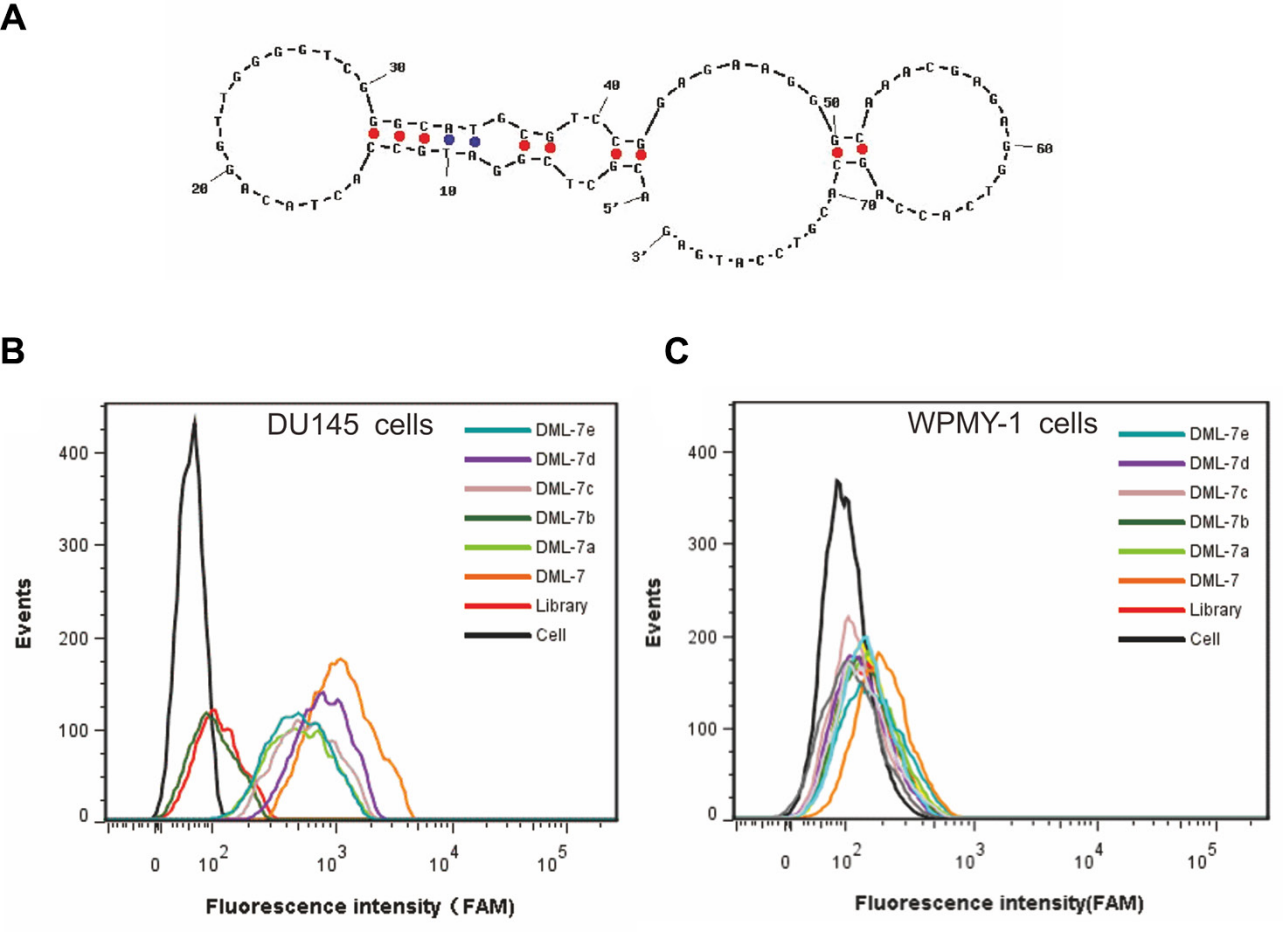
Binding characterization of DML-7 sequence (**A**) Secondary structure analysis of DML-7 predicted by NUPACK. (**B**) Binding of different truncted DML-7 to (B) DU145 and (**C**) WPMY-1 cells was assayed by flow cytometry.

According to secondary structure analysis of DML-7 predicted by NUPACK (Figure 5A), the sequence of DML-7 was truncated by gradually removing the nucleotides at the 5′ and 3′ termini. Five truncated sequences, designating DML-7a, DML-7b, DML-7c, DML-7d and DML-7e, were listed in Table 2. Their binding ability to DU145 cells was tested by flow cytometry. As shown in Figure 5B and 5C, all truncated sequences could bind to DU145, but not WPMY-1cells, indicating that they retained specific recognition ability to DU145 cells, even though sequences were truncated. Compared with untruncated DML-7, it should be noted that truncated sequences showed different degrees of decreased binding affinity to DU145 cells (Figure 5B). Among these, DML-7d showed the minimal influence on the binding ability. Thus, it can be concluded that both forward and reverse primers may be indispensable for successful DML-7 binding.

**Table 2:** Truncated sequnces from DML-7

Aptamer	Sequences
DML-7a	ATGCCACTACAGGTTGGGGTCGGGCATGCGTCCGGAGAAGGGCAAACGAGAGGTCACAGCAC
DML-7b	AGGTTGGGGTCGGGCATGCGTCCGGAGAAGGGCAAACGAGAGGT
DML-7c	ATGCCACTACAGGTTGGGGTCGGGCATGCGTCCGGAGAAGGGCAAACGAGAGGTCACCAGCA
DML-7d	CGGATGCCACTACAGGTTGGGGTCGGGCATGCGTCCGGAGAAGGGCAAACGAGAGGTCAC
DML-7e	CGGATGCCACTACAGGTTGGGGTCGGGCATGCGTCCGGAGAAGGGCAAACGAGAGGTCACCAGCA

### Specificity of DML-7 to other cell lines

**Figure 6:**
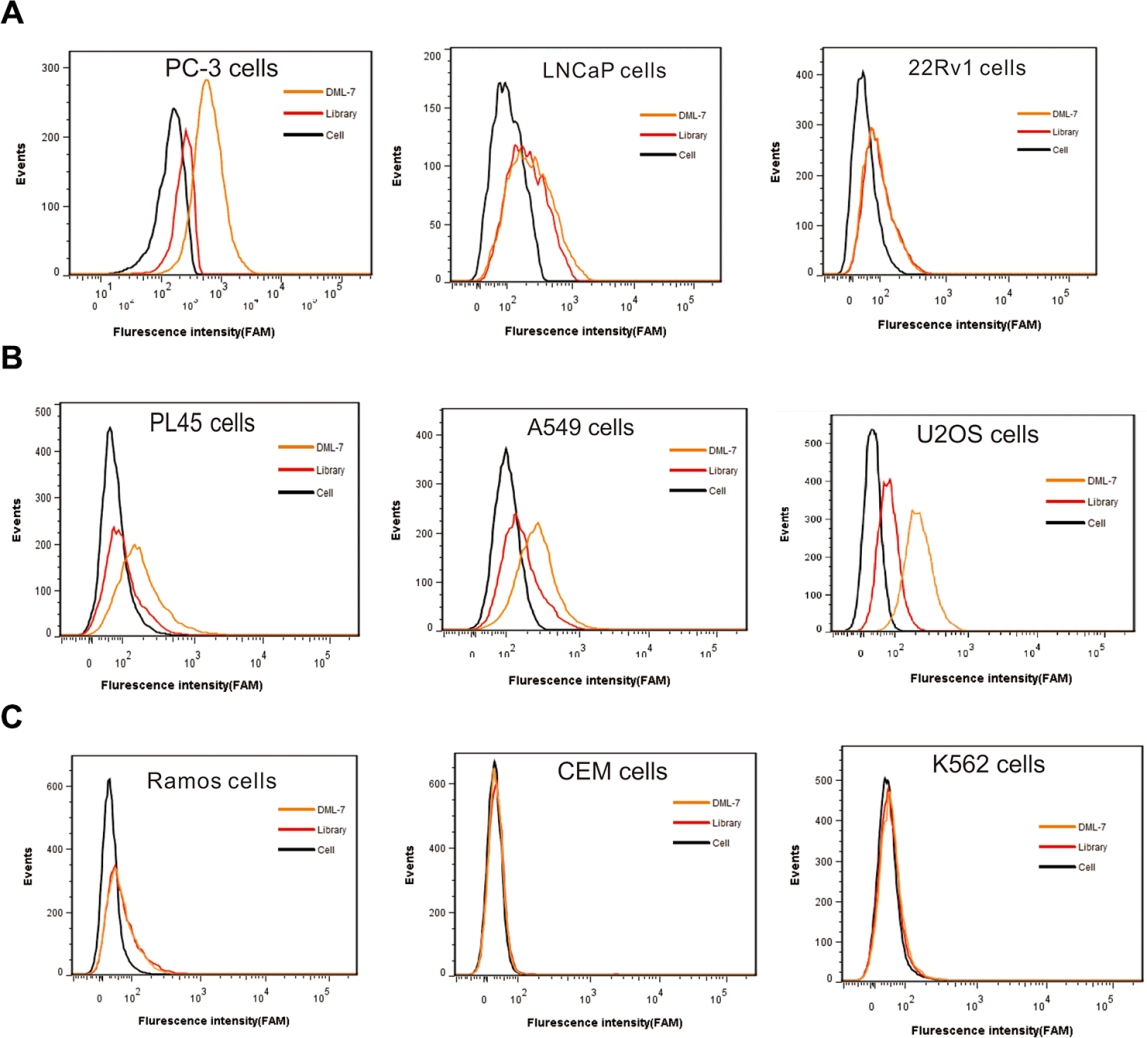
Specificity of DML-7 to other cell lines FAM-labeled DML-7 was incubated with (**A**) PCa cell lines with different origins, (**B**) epithelial cancer cell lines and (**C**) hematopoietic cell lines, respectively. Binding ability was analyzed by flow cytometry.

To further characterize DML-7 binding to other PCa cell lines, FAM-labeled DML-7 was incubated with AR-negative PC-3 cells and AR-positive LNCaP and 22Rv1 cells. Interestingly, we found that DML-7 only bound to AR-negative PC-3 cells, but not AR-positive LNCaP and 22Rv1 cells (Figure 6A). It is well known that AR plays a key role in the development, function and homeostasis of the prostate and is, therefore, closely associated with the development and progression of PCa [27]. Previous studies have revealed that epithelial AR may function as a suppressor of cell invasion and metastasis [28]. Human clinical data from PCa patients indicated that AR expression was significantly decreased in metastatic PCa as compared to primary PCa. Consistent with these studies, AR-negative PC-3 cells have high metastatic potential compared to AR-positive LNCaP and 22Rv1 cells, which have low, or no, metastatic potential [29]. Since DML-7 was generated by cell-SELEX using AR-negative DU145 cells with metastatic potential, it is reasonable to believe that the target protein of DML-7 may be correlated with aggressiveness or metastasis.

Although DML-7 was generated using PCa cells, the possibility of recognizing other types of cancer cells could not be excluded since its cell surface receptor is unknown. Therefore, the selectivity of DML-7 was further investigated with multiple cell lines of different origins, including human pancreatic adenocarcinoma cell line PL45, human lung adenocarcinoma cell line A549, human osteosarcoma cell line U2OS, human Burkitt's lymphoma cell line Ramos, human acute lymphoblastic leukemia cell line CEM and human chronic myelogenous leukemia cell line K562. Among all tested cell lines, we found that DML-7 bound PL45, A549 and U2OS cells, but not cell lines with suspension features, including Ramos, CEM and K562 (Figure 6B and 6C). These results indicate that DML-7 may have the potential to recognize cancer cell lines other than metastasis-specific DU145 and PC-3.

### Imaging of clinical PCa tissues with DML-7

**Figure 7:**
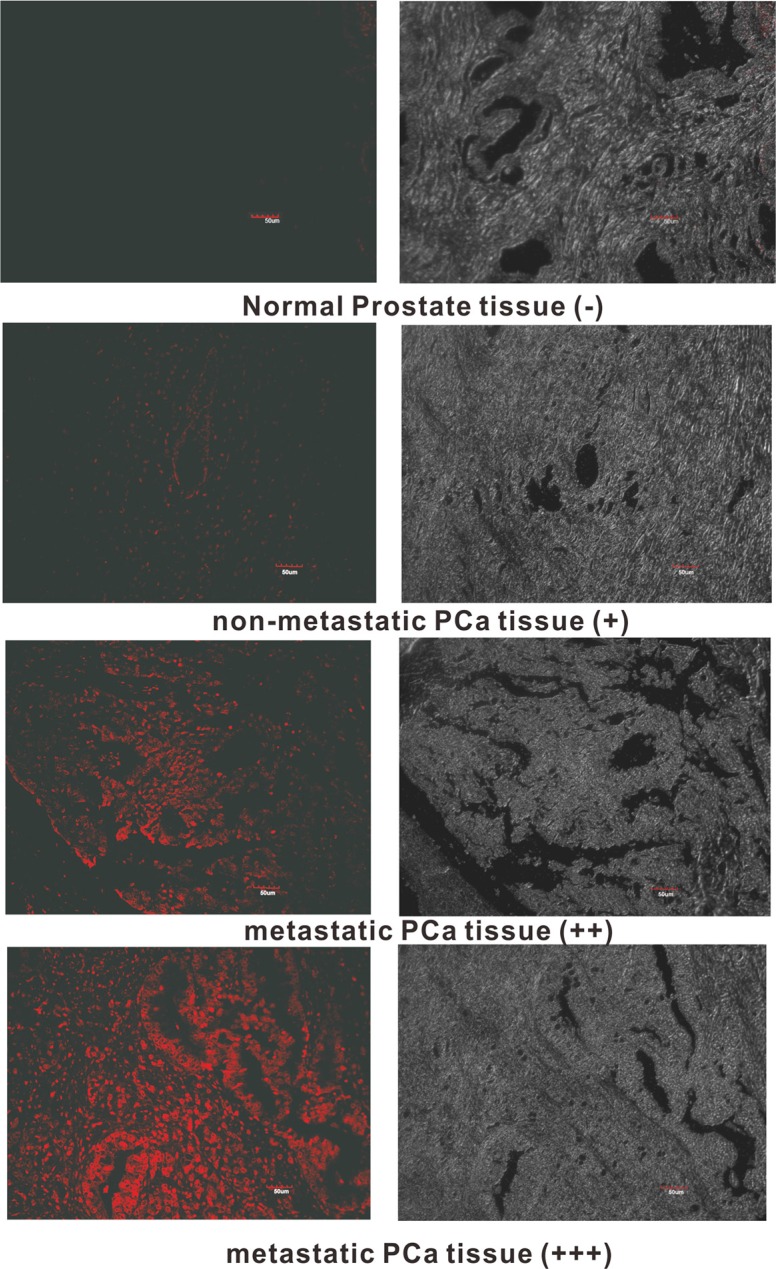
Fluorescence images of tissue section stained with FAM-labeled DML-7 Typical fluorescence image in normal prostate tissues (−, no fluorescence signal), nonmetastatic PCa tissues (+, weak fluorescence signal) and metastatic PCa tissues (++, moderate fluorescence signal; +++, strong fluorescence signal) was shown.

Since DML-7 shows high binding affinity for PCa cells with metastatic potential, we speculate that DML-7 may also have the ability to recognize clinical metastatic PCa tissues. To test this hypothesis, laser confocal fluorescence microscopy was used to image a prostate adenocarcinoma tissue microarray containing 40 cases of prostate adenocarcinoma and 8 normal prostate tissues with Cy5-labeled DML-7. As shown in Table 3 and Figure 7, all 8 normal prostate tissues and 85% (17/20) of nonmetastatic PCa tissues displayed weak or negative fluorescence signal. In contrast, all 20 cases of PCa tissues with metastasis exhibited strong or moderate fluorescence signal. These results indicate that DML-7 has strong binding and recognition ability for clinical metastatic PCa tissues.

**Table 3:** Assessment of clinical tissues stained with Cy-labeled DML-7

	Normal prostate tissues	Non-metastatic PCa tissues	Metastatic PCa tissues
−	4	1	0
+	4	16	0
++	0	3	12
+++	0	0	8

“−“: no fluorescence.

“+”: weak fluorescence intensity.

“++”: moderate fluorescence intensity.

“+++”: strong fluorescence intensity.

Although aptamer Wy-5a has already been generated against PC-3 cells [30], DML-7 varies from Wy-5a by its sequences, secondary structures and binding affinity. The difference derives from the fact that we adopted prostate cell line DU145 as target to obtain aptamer DML-7 by cell-based SELEX. An analysis of target type implies that the binding targets of DML-7 on the surface of PCa cells may be completely different from those of Wy-5a. Unlike Wy-5a, DML-7 binds two common metastatic prostate cell lines, DU145 and PC-3, but not low or nonmetastatic PCa cell lines LNCaP and 22Rv1. More importantly, the binding affinity of DU145 for clinical metastatic PCa tissues is closely associated with metastasis.

## MATERIALS AND METHODS

### Cell lines and cell culture

Human PCa cell lines DU145, LNCaP, 22Rv1, PC-3, and human prostatic stromal myofibroblast cell line WPMY-1 were obtained from the Chinese Academy of Sciences (CAS) cell bank (Shanghai, China). Human pancreatic ductal adenocarcinoma cell line PL45, human osteosarcoma cell line U2OS, human lung cancer cell line A549, human leukemic cell lines CEM and K562, and human Burkitt's lymphoma cell line Ramos were obtained from the American Type Culture Collection (ATCC). All cell lines were cultured at 37°C in a humid atmosphere with 5% CO_2_. DU145 cells were cultured in MEM medium supplemented with 10% fetal bovine serum (FBS, Hyclone) and 100 U/mL penicillin-streptomycin (pen-strep). PL45 cells were cultured in DMEM medium supplemented with 10% FBS and 100 U/mL pen-strep. WPMY-1 cells were cultured in DMEM medium supplemented with 5% FBS and 100 U/mL pen-strep. LNCaP, PC-3, 22Rv1, U2OS, A549, CEM, Ramos and K562 cells were cultured in RPMI-1640 medium supplemented with 10% FBS and 100 U/mL pen-strep.

### Buffer

Washing buffer was prepared by adding 5 mM of Mgcl_2_ and 4.5 g/L of glucose into Dulbecco's Phosphate-Buffered Saline (D-PBS). Binding buffer was prepared by adding 0.1 mg/mL of yeast tRNA and 1 mg/L of bovine serum albumin (BSA) to washing buffer.

### DNA synthesis and labeling

DNA used in the cell-SELEX was synthesized by Sangon Biotech Co. Ltd. (Shanghai, China). The ssDNA library was HPLC-purified and contained a randomized sequence of 40 nucleotides (nt) flanked by 20-nt sequences for primer annealing (5′-ACGCTCGGATGCCACTACAG-40nt-CTCATGGACGTGCTGGTGAC-3′). A FAM-labeled forward primer (5′-ACGCTCGGATGCCACTACAG-3′) and a biotinylated (Bio) reverse primer (5′-GTCACCA GCACGTCCATGAG-3′) were used in the PCR process for the synthesis of double-strand DNA (dsDNA) sequences.

### Cell-SELEX procedures

The Cell-SELEX process is nearly identical to that described previously with a few modifications [31, 32]. We preformed the selection process using DU145 cells as the target cells and WPMY-1 as the negative control cells. For each round of SELEX, DU145 and WPMY-1 cells were all cultured to 80%–90% confluence and kept in a good state. For the first and second round of SELEX, both cell lines were cultured in a 10 mm Petri dish. About 10 nmol initial ssDNA library were dissolved in 1.5 mL binding buffer. Before the process was carried out, the ssDNA pool was denatured by heating at 95°C for five minutes, followed by rapid cooling on ice to ensure that the DNA sequences would adopt the most favorable secondary structures. Then the DNA library was incubated with DU145 cells cultured in a 10 cm Petri dish at 4°C for 2 h. After discarding the supernatant solution, the cells were washed twice with washing buffer to remove unbound sequences. Afterwards, the cell-binding DNAs were harvested by heating at 95°C for 10 minutes and then centrifuging at 5000 rpm to remove cell debris. The harvested DNA was then used as a template and amplified by PCR with FAM-labeled forward primer and biotin-labeled reverse primers. PCR was performed by heating at 94°C for 3 minutes, 4–12 cycles of 30 s at 94°C, 30 s annealing at 55.2°C, and 30 s extension at 72°C, followed by 5 min at 72°C and 12°C thereafter. PCR Taq polymerase and dNTPs were obtained from Takara. FAM-labeled dsDNA was separated from the PCR solution by streptavidin-coated sepharose beads (GE Healthcare) and treated with 0.2 M of NAOH to get the ssDNA. Finally, after lyophilization and desalting, we obtained the ssDNA product for the next round of selection.

From the third round, negative WPMY-1 cells in a 60 mm culture dish with 90% confluence were incubated with the ssDNA evolved from the last round at 4°C for 30 min. Nonspecific binders were removed and applied on DU145 cells to enrich the specific sequences. The positive incubation time was shortened from 2 h to 1 h, and the negative incubation time was gradually increased from 30 min to 2 h to increase the stringency of the selection process. At the same time, washing times were also increased from 2 to 3 times. In total, 18 rounds of selection were performed before sequencing. The evolved ssDNA pool was sequenced using Illumina MiSeq (Sangon Biotech Co., Ltd. Shanghai, China).

### Flow cytometric analysis

Aptamer enrichment was monitored using flow cytometry. Target and negative cells were cultured in a 10mm dish with 80%~90% confluence, and cells were dissociated by 0.02 EDTA after washing with D-PBS twice. 3 × 10^5^ DU145 cells or WPMY-1 cells were incubated with 250 nM of FAM-labeled selected ssDNA pools and initial library pools in 100 ul of binding buffer at 4°C for 45 minutes. Before flow cytometric analysis (BD FACSVerse^™^ flow cytometer), each sample was washed three times with 500 uL of washing buffer and then filtered with 500 uL binding buffer. All experiments were repeated three times.

### Confocal microscopy imaging

Confocal microscopy imaging was employed to monitor the binding of aptamer to live cells. 1 × 10^5^ cells were seeded in a 35 mm glass bottom dish and cultured for about 24 h. The cells were then incubated with FITC- or Cy5-labeled aptamers at 4°C for 1 h after washing twice with cold washing buffer. After again washing twice with washing buffer, the cells were imaged by a FV1000-X81 confocal microscope (Olympus, Japan). The images were analyzed by FV10-ASW Version 3.1 software.

### Proteinase treatment of cells

Positive DU145 cells cultured to 80%–90% confluence were washed twice with D-PBS and then treated with 200 uL 0.25% trypsin or 0.1 mg/ml proteinase K for 3 minutes, followed by adding complete culture medium to inhibit proteinase activity. Cells were then washed with washing buffer, and the detached cells were collected. The cells were incubated with 250 nM of aptamers in 100 uL of binding buffer at 4°C for 45 minutes. After washing with washing buffer, all cell samples were tested by flow cytometry.

### Internalization analysis

Internalization analysis was conducted by confocal imaging. DU145 cells were incubated with 250 nM Cy5-labeled DML-7 or initial library in MEM medium at 4°C or 37°C for 2 h. Then, cells were washed three times with washing buffer before imaging.

### Affinity analysis

To test the affinity of aptamer DML-7, DU145 cells (3 × 10^5^) were incubated with different concentrations of aptamers and then analyzed by flow cytometry. Equilibrium dissociation constants (K_*d*_) of aptamers were obtained by flow cytometric assay by measuring the fluorescence intensity of specific binding by different aptamer concentration. Afterwards, results were fit as Y = B max X/(K_*d*_ + X), using Origin8 software.

### Using selected aptamer for human tumor tissue staining

A PCa tissue array (US Biomax, Inc) was preheated at 60°C for 2 h and then deparaffinized in xylene (15 min, twice). Tissue sections were then immersed in decreasing ethanol concentrations (100%, 95%, 90%, 80%, and 70%) at 5 min intervals. The hydrated tissues were pretreated in 0.01 M citrate buffer, pH 6.0, and heated in a pressure cooker for 20 min. Afterwards, tissue sections were blocked with precooled binding buffer, 20% FBS and 0.1 mg/ml Salmon Sperm DNA for 1 h at room temperature and incubated with 250 nM Cy5-labeled library or aptamer DML-7 at 4°C for 1 h. The signals were then detected using FV10-ASW v.3.1 software (Olympus) and displayed with an intensity scale ranging from 670 to 2940.
